# Research on the influence of bisphenol A on precocious puberty based on network toxicology and molecular dynamics simulation

**DOI:** 10.3389/fendo.2026.1850597

**Published:** 2026-07-22

**Authors:** Rui Nie, FeiQi Huang, Yu Zhan, Li Li, Yan Deng, Liang Tian, Jiao Luo, Bin Liang, Wenqing Yang

**Affiliations:** Department of Pediatrics, Sichuan Academy of Medical Sciences & Sichuan Provincial People’s Hospital, School of Medicine, University of Electronic Science and Technology of China, Chengdu, China

**Keywords:** bisphenol A, molecular dynamics simulation, network toxicology, precocious puberty, protox

## Abstract

**Purpose:**

Precocious puberty (PP) is a pediatric endocrine disorder. Bisphenol A (BPA) is an endocrine disruptor. This study aimed to computationally investigate BPA–associated PP targets and mechanisms via network toxicology and molecular dynamics.

**Methods:**

BPA information was obtained from PubChem. Target genes were collected from multiple databases, and PP-related genes were identified via GeneCards and Online Mendelian Inheritance in Man. ProTox was used to predict BPA toxicity. After gene set intersection, gene ontology (GO) and Kyoto Encyclopedia of Genes and Genomes (KEGG) analyses were performed (P < 0.05). A protein–protein interaction (PPI) network was constructed to identify key genes, and a BPA–key gene–pathway network was established. The subcellular localization of key genes was analyzed, followed by molecular docking (score ≤ −5 kcal/mol) and 100-ns GROMACS-based molecular dynamics simulation.

**Results:**

A total of 722 BPA targets and 1,126 potential PP targets were identified using multiple databases. Their intersection gave 99 potential target genes for BPA–induced PP, and further intersection with 16 toxic target genes of BPA resulted in six candidate genes. GO enrichment had 378 pathways, and KEGG enrichment had 18 pathways, with the top 10 being related to processes such as chemical carcinogenesis-receptor activation. The PPI network analysis revealed four key genes. Subcellular localization showed key gene product distribution. Molecular docking revealed a strong binding force, and molecular dynamics simulation further indicated that the complexes formed by BPA and these proteins were structurally stable.

**Conclusions:**

This study identified potential BPA–PP targets, providing a hypothesis-generating basis for future validation.

## 1 Introduction

Precocious puberty (PP) is a common pediatric endocrine disease (1). PP can cause progressive breast development in girls and increased testicular volume in boys, as well as premature epiphyseal closure (2, 3). In recent years, PP incidence has been on the rise, with girls predominating (2, 4). A recent multicenter prospective cohort study in China reported a PP incidence of 1.7% among children, with higher rates in girls (2.9%) than boys (1.1%) (5). The pathological mechanism of PP is complex. PP is classified as central PP (CPP), which is mainly caused by the premature activation of the hypothalamic–pituitary–gonadal (HPG) axis function, and as peripheral PP (PPP), which is mostly caused by abnormal secretion of sex hormones in parts such as the gonads or adrenal cortex, or secondary to a genetic disease, independent of gonadotropin secretion (6). The neuroendocrine regulation of pubertal onset involves a complex network of hypothalamic signals, including kisspeptin neurons (encoded by KISS1) acting on GnRH neurons via KISS1R, inhibitory regulation by MKRN3, and neurokinin B signaling through TAC3/TACR3, all of which converge to govern the timing of GnRH pulse generator activation (2). In addition, environmental and genetic factors play a role in PP occurrence and development (7, 8).

Bisphenol A (BPA) is a synthetic endocrine disruptor (EDC) that is sparingly soluble in water and more soluble in organic solvents (9). It is widely present in industrial manufacturing (such as polycarbonate plastics and electronic devices) (10, 11) and in personal care products (such as cosmetics, shampoo, and several food ingredients) (12). BPA has estrogen-like effects that may interfere with the HPG axis and potentially contribute to multiple adverse effects on human health (13). In the reproductive system, BPA exposure can cause developmental defects in infants and young children, disrupt fetal reproductive development during pregnancy, and even lead to fetal malformations (14). Radwan et al.’s (15) study provides evidence that exposure to BPA is associated with poorer semen quality. Human and animal studies suggest that BPA exposure is associated with altered pubertal timing. In Chinese girls, urinary BPA has been associated with pubertal changes (16) and was detected in 11.3% of PP cases with a higher prevalence than controls (17). BPA substitutes (BPS, TBBPA, and BPFL) have also been linked to PP risk (18). In rats, neonatal BPA exposure promoted puberty by upregulating hypothalamic Kiss1/GnRH1 expression, suggesting the possible involvement of the kisspeptin pathway (19). Although many countries have implemented control measures, amid increasingly complex application scenarios and potential risks, in-depth research on its potential mechanism of action and exposure risks is warranted to address BPA–induced public health and environmental challenges.

This study aims to computationally explore the potential targets and pathways through which BPA may be associated with PP in order to generate hypotheses for future experimental investigations.

## 2 Materials and methods

### 2.1 Acquisition of potential target genes for BPA–induced PP

**Figure 1:**
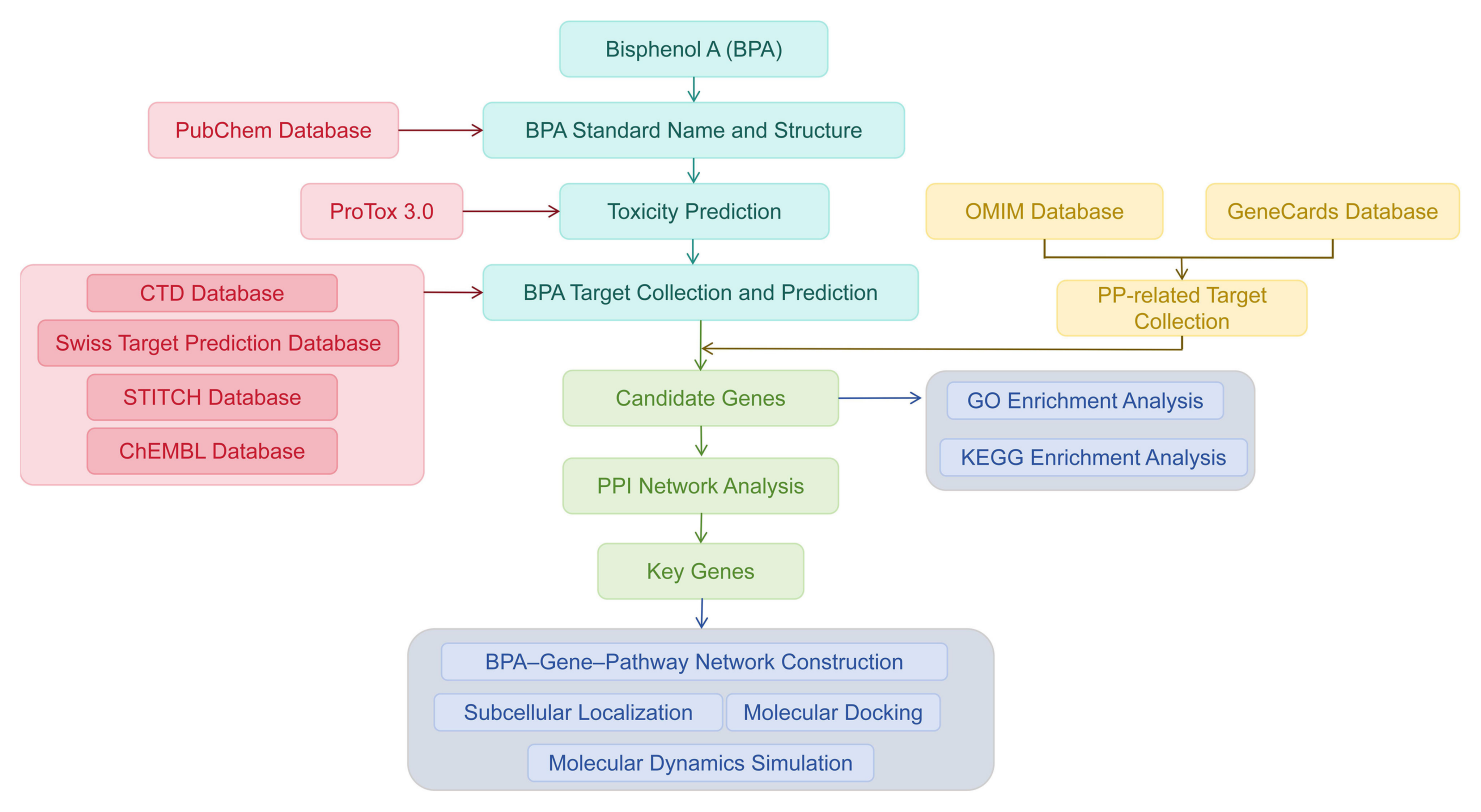
Flowchart of the study design. The diagram summarizes the BPA target collection (PubChem, ChEMBL, STITCH, SwissTargetPrediction, CTD), PP-related targets (GeneCards, OMIM), toxicity prediction (ProTox 3.0), candidate gene identification via intersection, followed by GO/KEGG enrichment, PPI network construction, and molecular docking and dynamics simulations of key genes. BPA, bisphenol A; PP, precocious puberty; GO, Gene Ontology; KEGG, Kyoto Encyclopedia of Genes and Genomes; PPI, protein–protein interaction; CTD, Comparative Toxicogenomics Database; OMIM, Online Mendelian Inheritance in Man.

**Figure 1** shows the workflow. Briefly, BPA- and PP-related targets were retrieved from multiple databases, and overlapping genes were identified. These candidates underwent enrichment analysis and PPI network construction, and were further validated by molecular docking and molecular dynamics simulations.

The chemical structure, canonical simplified molecular input line entry system (SMILES), and molecular weight of “BPA” were retrieved from the PubChem database (https://pubchem.ncbi.nlm.nih.gov/).

The data were processed using the “tidyverse” package (V 2.0.0) (20) and the “dplyr” package (V 1.1.4) (https://CRAN.R-project.org/package=dplyr). Potential BPA targets (Homo sapiens) were retrieved from ChEMBL (https://www.ebi.ac.uk/chembl/), the Search Tool for Interacting Chemicals database (STITCH, http://stitch.embl.de, with an interaction score > 0.4), the Swiss Target Prediction database (http://swisstargetprediction.ch/), and the Chemical Toxicity Database (CTD, https://ctdbase.org/, with the number of references > 5), then merged and deduplicated. Gene names were standardized using the Universal Protein Resource database (UniProt, https://www.uniprot.org/). PP-related targets were obtained from GeneCards (https://www.genecards.org/, median threshold) and the Online Mendelian Inheritance in Man database (OMIM, https://omim.org/), and similarly merged after deduplication. The intersection of BPA and PP targets was identified using the “ggvenn” package (V 0.1.9) (https://CRAN.R-project.org/package=ggvenn) and defined as putative BPA–associated PP targets.

### 2.2 Toxicity prediction

BPA toxicity was predicted using the ProTox database (V 3.0) (https://tox.charite.de). Structural similarity analysis was performed against reference compounds in the database, and the relative activity across toxicity categories was evaluated. Predicted toxicity–related targets were retrieved and standardized using UniProt to obtain BPA toxicity target genes.

### 2.3 Acquisition and functional analysis of candidate genes

The intersection of BPA–induced PP targets and BPA toxicity targets was identified using the “ggvenn” package (V 0.1.9), and overlapping genes were defined as candidate genes.

To explore the biological functions and signaling pathways involved in PP pathogenesis for the candidate genes, the gene ontology (GO) functional enrichment analysis, (biological process (BP), cellular component (CC), and molecular function (MF); P < 0.05)) and the Kyoto Encyclopedia of Genes and Genomes (KEGG) enrichment analysis (P < 0.05) were performed using the “clusterProfiler” package (V 4.7.1.003) (21) and the “org.Hs.eg.db” package (Version 3.16.0) (22). Results were visualized using the “ggplot2” package (V 3.4.4) (23).

### 2.4 Acquisition and functional analysis of key genes

The STRING database(http://www.string-db.org/, confidence ≥ 0.7, Homo sapiens) was used to construct a protein–protein interaction (PPI) network, and key genes were identified based on network degree centrality (presence vs. absence of interaction edges). No topological ranking (e.g., cytoHubba, centrality metrics) was applied, as this study prioritized toxicologically relevant targets from database intersections rather than hub genes; thus, STRING-based filtering was considered sufficient.

A BPA–key gene–KEGG pathway network was constructed using the “ggsankey” package (V 0.0.9) (https://github.com/davidsjoberg/ggsankey). Subcellular localization of key genes was predicted by retrieving protein sequences from NCBI (https://www.ncbi.nlm.nih.gov/), and analyzing them using the mRNALocater database (http://bio-bigdata.cn/).

### 2.5 Molecular docking and molecular dynamics simulation of key genes

The three-dimensional structure of the ligand BPA was downloaded from the PubChem database (https://pubchem.ncbi.nlm.nih.gov/), and the protein structures encoded by the key genes were downloaded from the rcsb protein databank database (PDB) (https://www.rcsb.org/). Molecular docking was performed using the CB-Dock database (https://cadd.labshare.cn/cb-dock/php/manual.php) (score ≤ −5 kcal/mol).

Molecular docking between BPA and proteins encoded by key genes was performed using CB-Dock, and 100 ns molecular dynamics simulation was conducted using GROMACS software (V 2024.2). The “AMBER14SB” force field and the “AMNER GAFF” force field were used to generate the parameters and topology files of proteins and small-molecule ligands, respectively. The systems were solvated with 0.15 M Na^+^ and Cl^−^, energy-minimized using the steepest descent, and equilibrated under NVT (300 K, 100 ps) and NPT (1 bar, 100 ps) ensembles. System stability and structural dynamics were assessed using root mean square deviation (RMSD), root mean square fluctuation (RMSF), and radius of gyration (Rg) analyses. To further characterize BPA–protein interactions, hydrogen bond occupancy was calculated using GROMACS gmx hbond (0.35 nm distance, 30° angle cutoff) over the 0–100 ns trajectory. Solvent-accessible surface area (SASA) was computed using gmx sasa with a 0.14 nm probe radius. Principal component analysis (PCA) of Cα atoms was performed using gmx covar and gmx anaeig, and free energy landscapes were constructed based on projections onto PC1 and PC2. Binding free energies were estimated using gmx_MMPBSA from 30 evenly sampled frames (0–100 ns, ~3.3 ns interval), without entropy contribution; thus, values represent enthalpic binding energy (ΔH).

## 3 Results

### 3.1 Molecular characteristics of BPA and the identification of potential targets associated with PP

**Figure 2:**
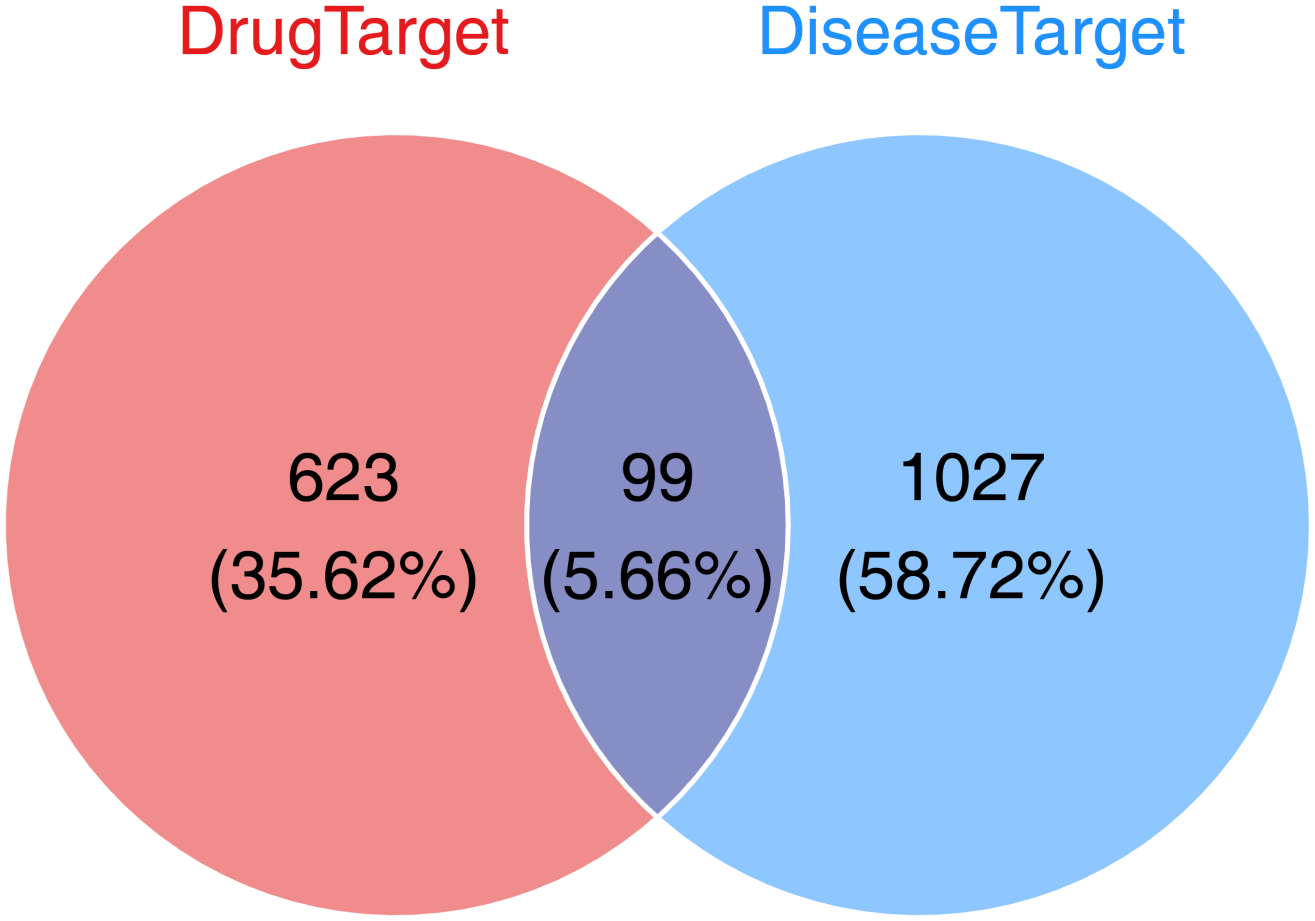
Venn diagram illustrating the overlap between BPA-related targets and PP-related targets. The intersection identifies 99 potential target genes for BPA–induced PP. BPA, bisphenol A; PP, precocious puberty.

In this study, the SMILES notation of BPA was CC(C)(C1=CC=C(C=C1)O)C2=CC=C(C=C2)O, the chemical formula was C15H16O2, and the molecular weight was 228.29 g/mol. First, 615 targets were obtained from the ChEMBL database; 10 targets were obtained from the STITCH database; 108 targets were obtained from the Swiss Target Prediction database; and 53 targets were obtained from the CTD database. After the integration and removal of duplicates, 722 potential targets of BPA were obtained **Supplementary Table 1**). Second, the 1,002 and 380 targets were retrieved from the GeneCards and OMIM databases, respectively. After the integration and removal of duplicates, 1,126 potential targets of PP were obtained (**Supplementary Table 2**). Finally, by taking the intersection, 99 common potential targets between BPA and PP were identified, which were the potential target genes for BPA–induced PP (**Figure 2**; **Supplementary Table 3**).

### 3.2 Predicting BPA toxicity

**Figure 3:**
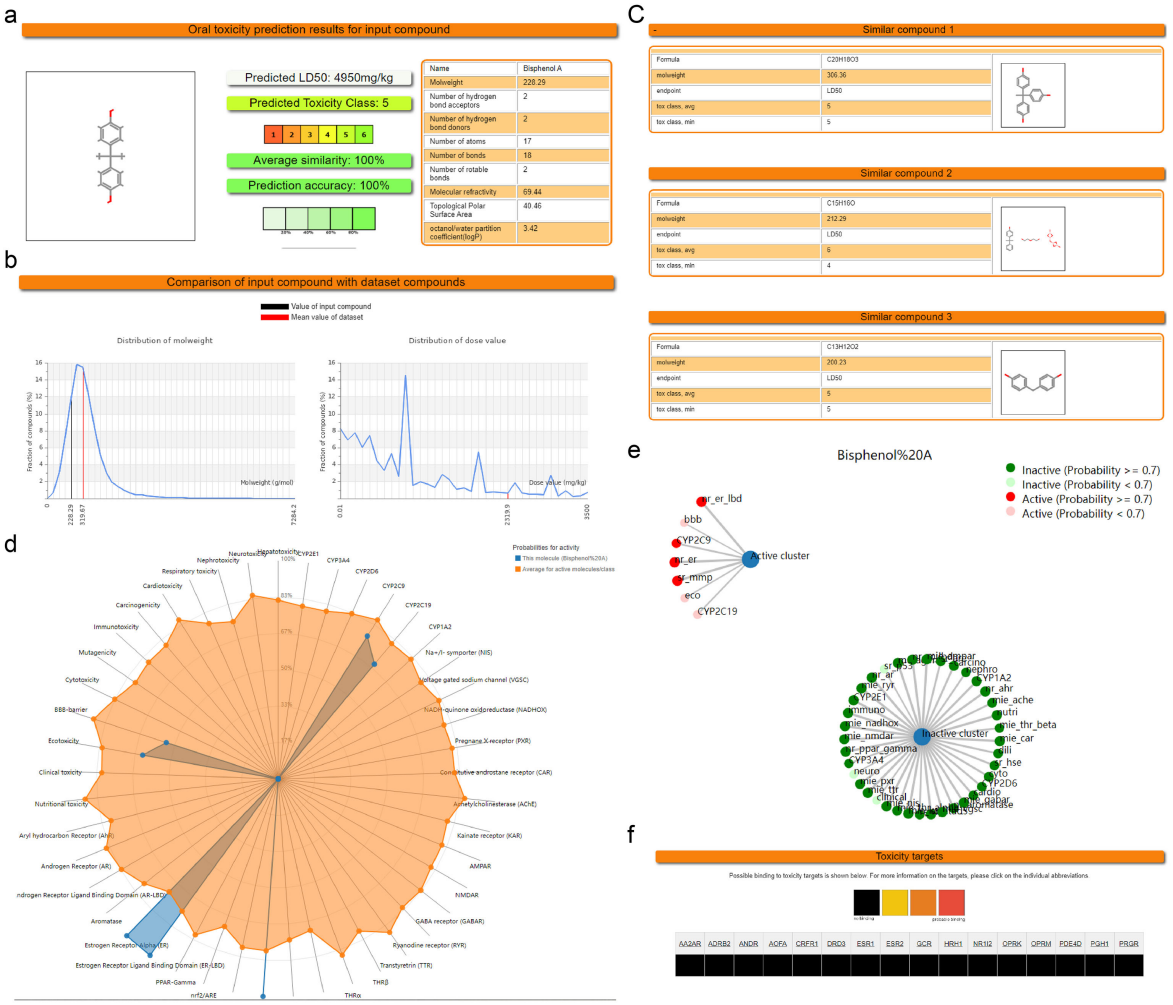
Predicting the toxicity of BPA. **(a)** Information on the acute toxicity of BPA. **(b)** BPA is combined with the average dataset comparison of objects. Black: Value of the input compound. Red: Mean value of the dataset. **(c)** Three compounds most similar to BPA in the dataset. **(d)** Radar map of BPA toxicity activity probability. Orange: Average of active molecules/class; Blue: This molecule (Bisphenol%20A) **(e)** Relationship between BPA activity and different molecular targets. Connecting lines represent interactions. The darker the color, the greater the probability. Different colors represent different bindings. **(f)** The toxic targets predicted by BPA. BPA, bisphenol A; LD50, median lethal dose; ER-LBD, Estrogen Receptor Ligand Binding Domain; ER, estrogen receptor; NIS, Na^+^/I^-^ symporter; CYP2E1, cytochrome P450 2E1.

The results of the toxicity prediction showed that the acute oral toxicity of BPA was relatively low. Its LD50 value was 4,950 mg/kg, and the toxicity classification level was grade 5. Moreover, the model’s average similarity and prediction accuracy were both 100%, indicating a high accuracy of the prediction results (**Figure 3a**).

Meanwhile, the molecular weight and dose values of BPA were lower than the average values of the drugs in the dataset (**Figure 3b**). The three compounds most similar to BPA in the dataset were C20H18O3, C15H16O, and C13H12O2 (**Figure 3c**). The molecular weight and dose-value distributions of BPA were compared with those of compounds in the dataset, and structurally similar compounds were also identified. These analyses helped clarify the similarities and differences between BPA and other compounds and provided a basis for evaluating its characteristics, potential toxicity, and performance in relevant research or application settings.

The toxicity radar charts showed the prediction categories and confidence scores of BPA in terms of organ toxicity, toxicological endpoints, toxicological pathways, etc. The radar chart results showed that the activity probabilities of BPA were relatively high in “Estrogen Receptor Ligand Binding Domain (ER-LBD)” and “Estrogen Receptor Alpha (ER)”. Additionally, the charts also showed that the activity probability of BPA in “CYP2E1” was 80% and that in “Na^+^/I^−^ symporter (NIS)” was 79% (**Figure 3d**).

The activity relationships between BPA and different molecular targets were presented through another radar chart, based on which active and inactive clusters were identified. In the active clusters (dark red), targets such as nr_er_lbd, CYP2C9, and sr_mmp were included. The activity probabilities of BPA with these targets were at least 0.7. For targets such as CYP2E1, CYP2D6, and CYP1A2 in the inactive clusters (dark green), the activity probabilities of BPA with them were also at least 0.7, but they played relatively minor roles in the biological effects of BPA. In addition, light green indicated the inactive state, where the activity probability of BPA with targets was below 0.7. For targets such as neuro-related targets, the predicted probability of BPA binding and inducing significant biological effects was low, suggesting limited involvement in the physiological or pathological processes associated with these targets. Light red indicated a low-activity state, in which the predicted activity probability between BPA and the targets was <0.7. For targets such as ECO and CYP2C19, BPA showed predicted activity probabilities below 0.7, suggesting weak but possible biological interactions with these targets (**Figure 3e**). In addition, 16 predicted toxic targets of BPA were identified. After standardization through UniPort, the target genes obtained were *ADORA2A*, *ADRB2*, *AR*, *MAOA*, *CRHR1*, *DRD3*, *ESR1*, *ESR2*, *HRH1*, *NR1I2*, *OPRK1*, *OPRM1*, *PDE4D*, *PTGS1*, and *PGR* (**Figure 3f**). These results were helpful for comprehensively understanding BPA toxic characteristics.

### 3.3 Acquisition of candidate genes and pathways related to BPA and PP

**Figure 4:**
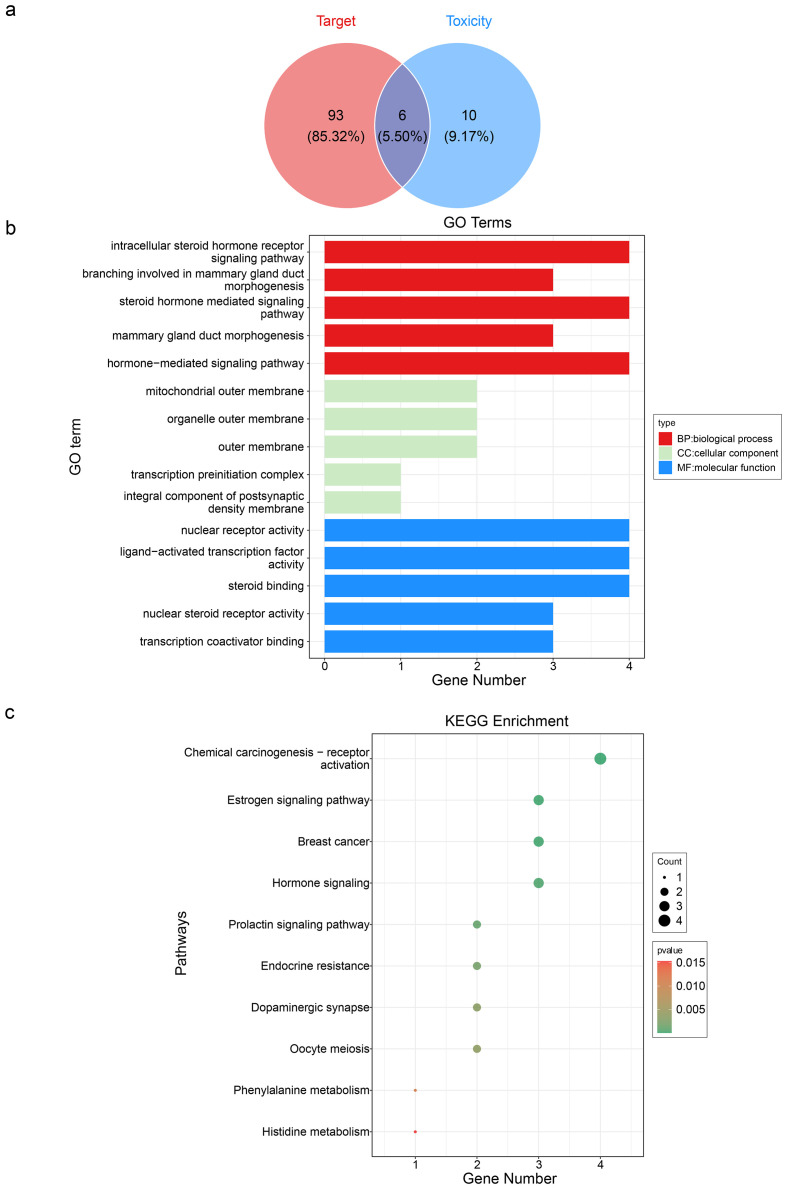
Acquisition of candidate genes and pathways related to BPA and PP. **(a)** Venn diagram of the candidate genes. **(b)** GO enrichment analysis of candidate targets. The color of the column distinguishes different GO categories, and the length of the column indicates that GO. The term “enriched gene quantity” indicates that the longer the column, the more enriched genes there are. The text identifiers on the column are enriched to the path names, and the column at the bottom of the column. Identify the genes that are specifically enriched in this pathway. The horizontal coordinate represents the number of genes enriched in the relevant pathways. **(c)** KEGG pathway enrichment analysis of candidate targets. The size of the bubble plot indicates the number of genes enriched in this pathway. The larger the bubble plot, the more genes are enriched. The color saturation of the bubble corresponded to P values. BPA, bisphenol A; PP, precocious puberty; GO, Gene Ontology; KEGG, Kyoto Encyclopedia of Genes and Genomes; BP, biological process; CC, cellular component; MF, molecular function.

The intersection of the 99 potential target genes of BPA–induced PP and the 16 toxic target genes of BPA was taken, and a total of six candidate genes were obtained, which were *MAOA*, *ESR1*, *ESR2*, *PGR*, *AR*, and *DRD3* (**Figure 4a**).

Subsequently, a GO enrichment analysis was conducted on the candidate genes, yielding a total of 378 results, including 333 BP, 16 CC, and 29 MF. The top five pathways with the most significant gene enrichment in each part were selected for display. When considering BP, the top five most prominent ones identified included the intracellular steroid hormone receptor signaling pathway, branching involved in mammary gland duct morphogenesis, steroid hormone-mediated signaling pathway, mammary gland duct morphogenesis, and hormone-mediated signaling pathway. In terms of CC, the top five most significant ones identified were the mitochondrial outer membrane, organelle outer membrane, outer membrane, transcription preinitiation complex, and integral component of the postsynaptic density membrane. In terms of MF, the top five were nuclear receptor activity, ligand-activated transcription factor activity, steroid binding, nuclear steroid receptor activity, and transcription coactivator binding (**Figure 4b**).

Meanwhile, a KEGG functional enrichment analysis was performed on the candidate genes, resulting in 18 related pathways. The top 10 pathways with the most significant enrichment results were presented (**Figure 4c**; **Supplementary Table 4**).

### 3.4 Screening key genes in the PPI network and constructing the BPA–key gene–pathway network

**Figure 5:**
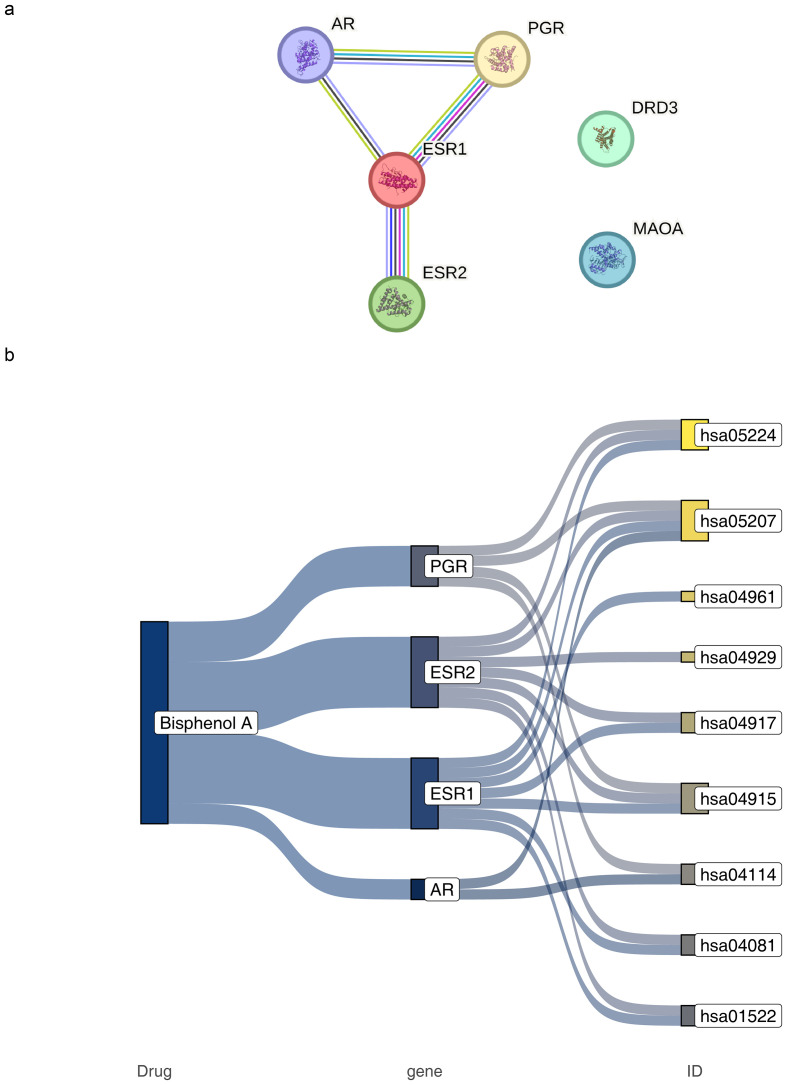
Screen key genes in the PPI network and construct the BPA–key gene–pathway network. **(a)**. STRING analysis network diagram of candidate genes. Illustrations with lines in the figure have pairs of protein interaction relationships. **(b)** BPA–Key Gene–Pathway network diagram. PPI, protein–protein interaction; BPA, bisphenol A.

The PPI network presented a topological structure with six nodes and four edges. DRD3 and MAOA manifested as isolated targets in the network. To clarify the role pathway of BPA target genes in PP occurrence and development, this study excluded the interference of the above-mentioned isolated targets. Based on an interaction score threshold of ≥0.7, *AR*, *PGR*, *ESR1*, and *ESR2* were finally established as key genes. This result provided clear core targets for the detailed exploration of PP pathogenesis (**Figure 5a**). A lower STRING threshold (0.4) was also tested, with *AR*, *ESR1*, *ESR2*, and *PGR* remaining stable across settings, indicating robust screening results.

In this study, the BPA–key gene–pathway network constructed covered one drug BPA, four key genes (*AR*, *ESR1*, *ESR2*, *PGR*) and 9 KEGG pathways. The network revealed that BPA and the four key genes were co-enriched in pathways including breast cancer and chemical carcinogenesis-receptor activation (**Figure 5b**).

### 3.5 The subcellular distribution of the key genes was determined

**Figure 6:**
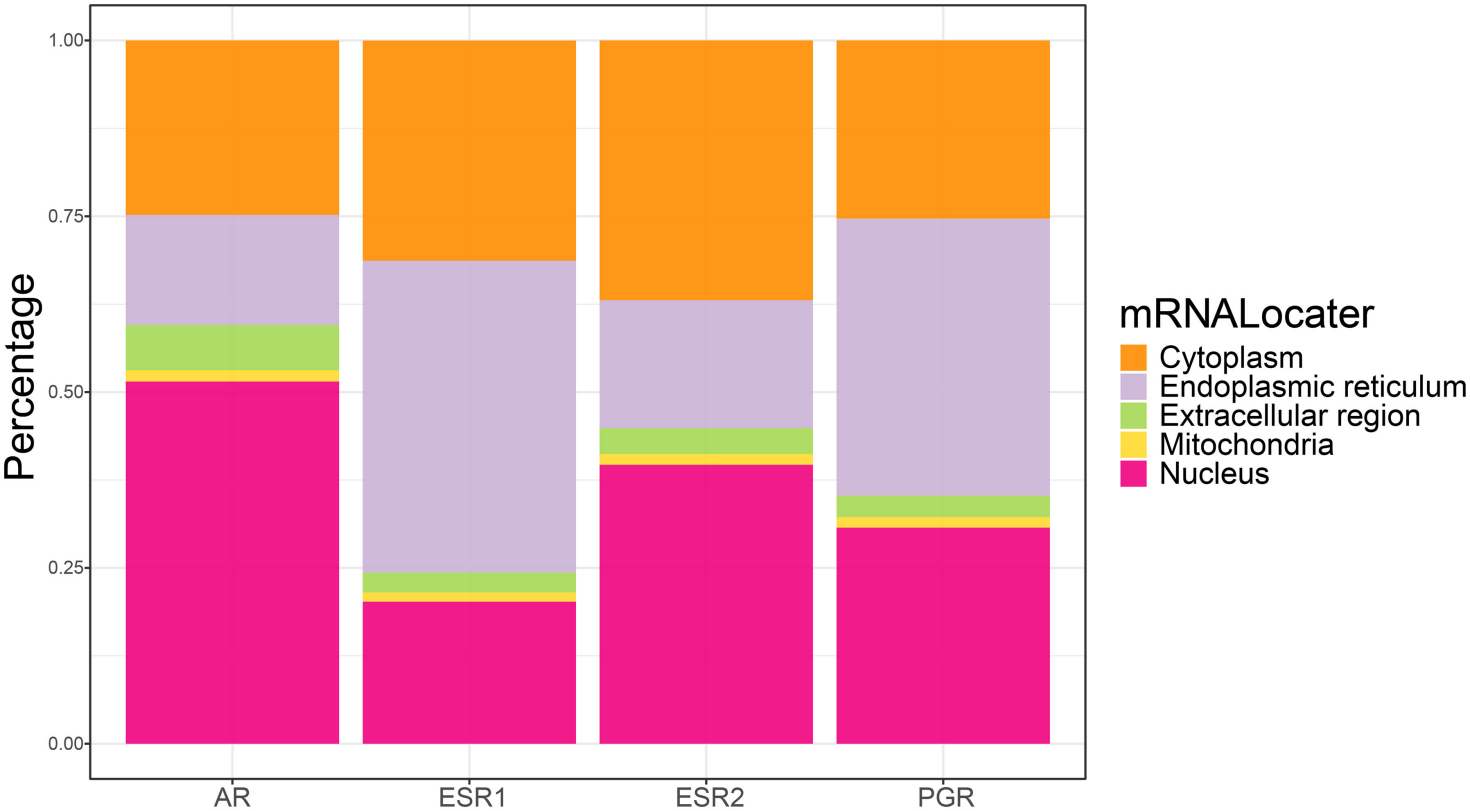
Subcellular localization maps of key genes. Different colors represent different positions.

The subcellular localization of the key genes showed that *AR* and *ESR2* products were mainly distributed in the nucleus; *ESR1* and *PGR* products were mainly distributed in the endoplasmic reticulum (**Figure 6**).

### 3.6 BPA exhibits a significant binding affinity toward proteins encoded by key genes

**Figure 7:**
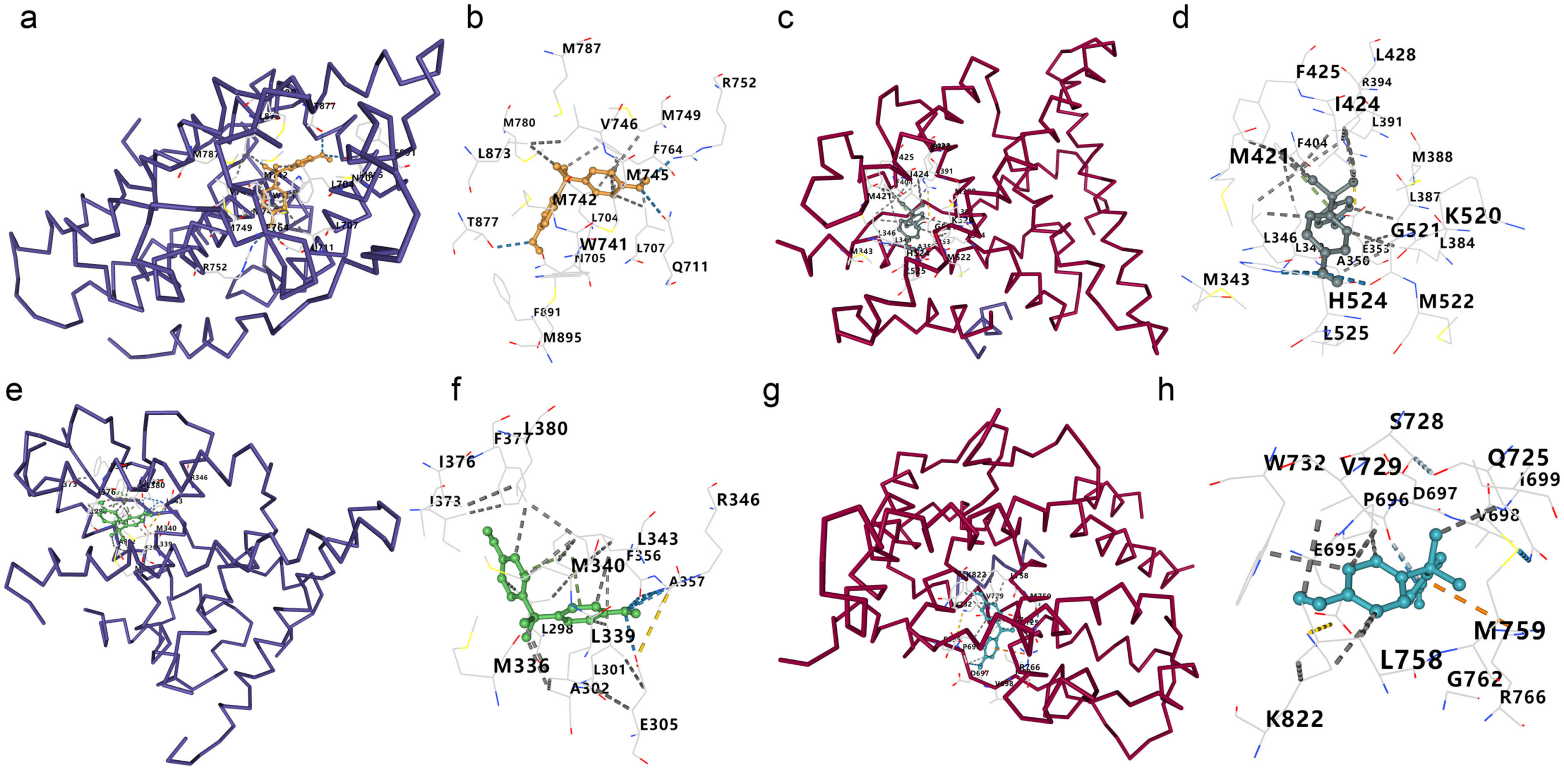
Molecular docking of BPA with proteins encoded by key genes **(a, b)** BPA and *AR*; **(c, d)** BPA and *ESR1*; **(e, f)** BPA and *ESR2*; **(g, h)** BPA and *PGR*. The left image is the overall picture, while the right image is an enlarged view of the action position figure (Hidden protein structure). The middle number represents the residue in the connection. BPA, bisphenol A; AR, androgen receptor; ESR1, estrogen receptor alpha; ESR2, estrogen receptor beta; PGR, progesterone receptor; PDB, Protein Data Bank.

Molecular docking was conducted using protein structures retrieved from the PDB (AR: 1E3G; ESR1: 1X7R; ESR2: 1NDE; PGR: 2OVM). The docking results demonstrated that all four BPA–protein complexes exhibited binding affinities below the −5.0 kcal/mol threshold, BPA and *AR* was the most stable (−8.5 kcal/mol), followed by *ESR1* (−8.2 kcal/mol), *ESR2* (−7.9 kcal/mol), and *PGR* (−7.6 kcal/mol) (**Table 1**, **Figures 7a–h**). These results demonstrated a strong binding affinity between BPA and the proteins encoded by all four key genes.

**Table 1:** Molecular docking results between BPA and each target protein.

Gene	Pro ID	Molecule	Vina score(kcal/mol)
*AR*	P10275	Bisphenol A	−8.5
*ESR1*	P03372	Bisphenol A	−8.2
*ESR2*	Q92731	Bisphenol A	−7.9
*PGR*	P06401	Bisphenol A	−7.6

### 3.7 BPA binds stably with proteins encoded by key genes

**Figure 8:**
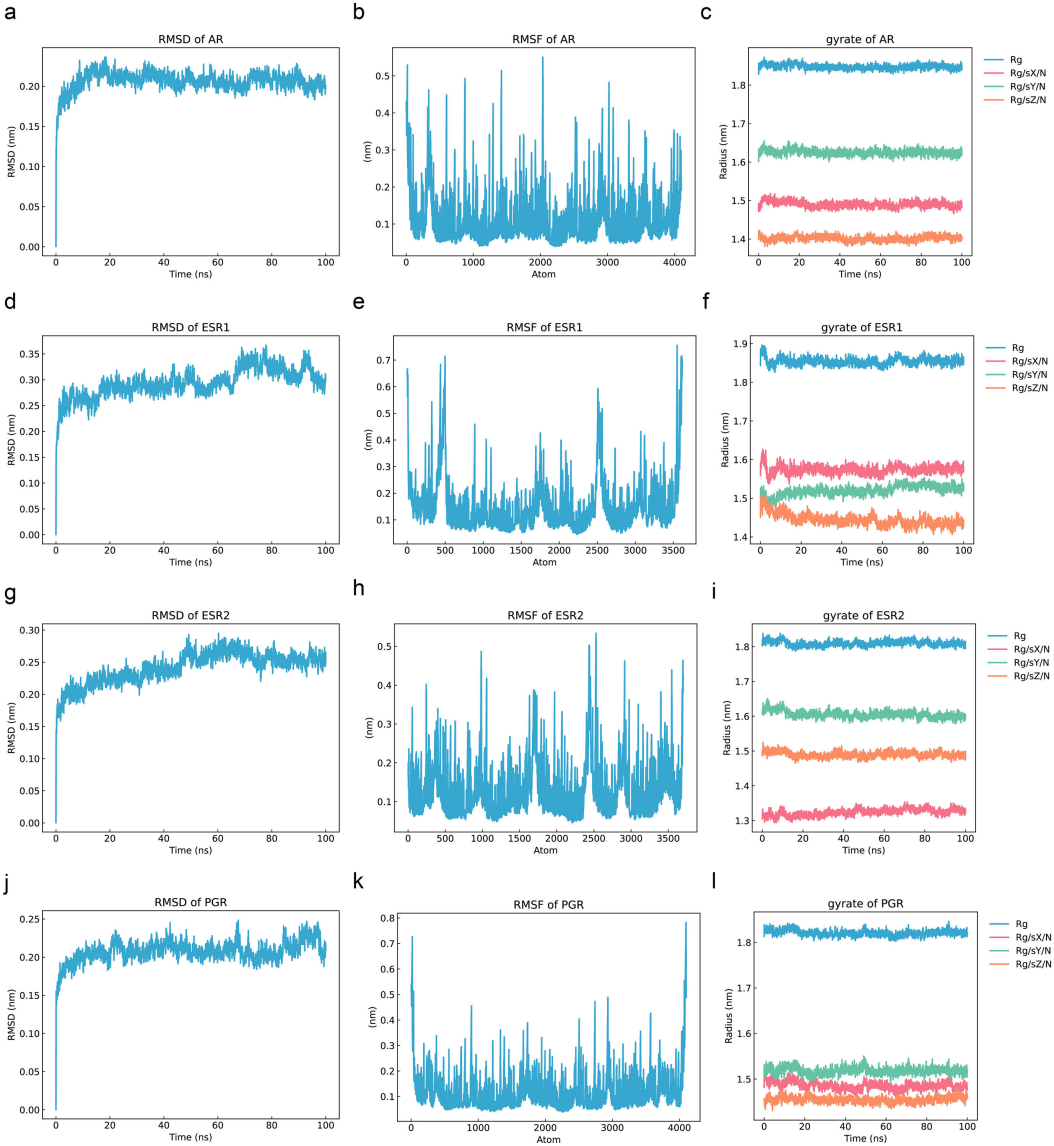
Molecular dynamics simulation results for BPA–protein complexes over 100 ns. **(a)** RMSD of *AR*; **(b)** RMSF of *AR*; **(c)** gyrate of *AR*; **(d)** RMSD of *ESR1*; **(e)** RMSF of *ESR1*; **(f)** gyrate of *ESR1*; **(g)** RMSD of *ESR2*; **(h)** RMSF of *ESR2*; **(i)** gyrate of *ESR2*; **(j)** RMSD of *PGR*; **(k)** RMSF of *PGR*; **(l)** gyrate of *PGR.* BPA, bisphenol A; AR, androgen receptor; ESR1, estrogen receptor alpha; ESR2, estrogen receptor beta; PGR, progesterone receptor; RMSD, root mean square deviation; RMSF, root mean square fluctuation.

The results of the molecular dynamics simulation of the key gene *AR* showed that in terms of RMSD analysis, most of the protein RMSD values in the 100-ns kinetic simulation were between 0.15 and 0.25 nm. This indicated that from 20 ns to 100 ns, the change in the position of the protein conformation compared with the initial conformation was small, the overall state was stable, and the simulation reached a stable state (**Figure 8a**). The RMSF analysis demonstrated that during the entire simulation duration, the atoms in the protein had good flexibility, were stably bound to the small-molecule drug ligands, and the degree of atomic motion was suitable for maintaining the binding (**Figure 8b**). The analysis of the radius of gyration revealed that during the simulation time, the radius of gyration of the protein peptide chain in the X-axis, Y-axis, and Z-axis directions, and overall all showed that the degree of peptide chain looseness was stable, with no significant change in the peptide chain compactness, further proving protein stability (**Figure 8c**).

The results of the molecular dynamics simulation of the key gene *ESR1* showed that, in terms of RMSD analysis, within the 100-ns simulation time, the RMSD value of *ESR1* started low, then gradually increased and fluctuated between 0.25 and 0.35 nm. This indicated that during the simulation process, the ESR1 conformation had certain changes compared with the initial conformation but still maintained a relatively stable state within the fluctuation range (**Figure 8d**). The RMSF analysis showed that the RMSF values of different atoms in *ESR1* differed significantly. The RMSF values of some atoms were relatively high, indicating that these atoms had relatively intense motion and greater flexibility during the simulation, while the RMSF values of some atoms were relatively low, indicating that their flexibility was smaller and their motion was relatively stable. Overall, this reflected the difference in atomic flexibility in *ESR1* (**Figure 8e**). In the radius of gyration, within 0–100 ns, the curves of the radius of gyration representing the overall radius of gyration Rg and those in the X-axis, Y-axis, and Z-axis directions fluctuated but remained within a relatively stable range, indicating that the degree of looseness of the *ESR1* protein peptide chain was stable and that no significant change existed in the structural compactness (**Figure 8f**).

The results of the molecular dynamics simulation of the key gene *ESR2* showed that, in the RMSD graph, within the 0–100 ns simulation time, the RMSD value of *ESR2* started low, then gradually increased and fluctuated between 0.20 and 0.30 nm. This indicated that during the simulation process, the *ESR2* conformation had certain changes compared with the initial conformation but still maintained a relatively stable state within the fluctuation range (**Figure 8g**). The RMSF graph demonstrated that the RMSF values of the different atoms differed significantly. The RMSF values of some atoms were relatively high, which meant that these atoms moved vigorously and had high flexibility during the simulation, while the RMSF values of some atoms were relatively low, indicating that they had low flexibility and relatively stable movement, reflecting the difference in the atomic flexibility of *ESR2* (**Figure 8h**). In the radius of gyration graph, within 0–100 ns, although the curves of the radius of gyration representing the overall radius of gyration Rg and those in the X-axis, Y-axis, and Z-axis directions fluctuated, they were all within a relatively stable range, indicating that the degree of looseness of the *ESR2* protein peptide chain was stable and that no significant change existed in the structural compactness (**Figure 8i**).

The results of the molecular dynamics simulation of the key gene *PGR* showed that, in the RMSD graph, within the 0–100 ns simulation time, the RMSD value of *PGR* started extremely low, then rapidly increased and fluctuated between 0.15 and 0.25 nm. This indicated that during the simulation process, the *PGR* conformation had certain changes compared with the initial conformation but maintained a relatively stable state within the fluctuation range (**Figure 8j**). The RMSF graph demonstrated that the RMSF values of different atoms in the *PGR* differed significantly. The RMSF values of some atoms were extremely high, meaning that these atoms moved extremely vigorously and had extremely high flexibility during the simulation, while the RMSF values of some atoms were relatively low, indicating that their flexibility was small and the movement was relatively stable, reflecting the obvious difference in the atomic flexibility of *PGR* (**Figure 8k**). In the radius of gyration graph, within 0–100 ns, although the curves of the radius of gyration representing the overall radius of gyration Rg and those in the X-axis, Y-axis, and Z-axis directions fluctuated, they were all within a relatively stable range, indicating that the degree of looseness of the *PGR* protein peptide chain was stable and that no significant change existed in the structural compactness (**Figure 8l**). Collectively, these results demonstrated that the complexes formed by BPA with *AR*, *ESR1*, *ESR2*, and *PGR* maintained structural stability throughout the 100-ns simulation.

### 3.8 BPA forms energetically favorable and dynamically stable complexes with proteins encoded by key genes

**Figure 9:**
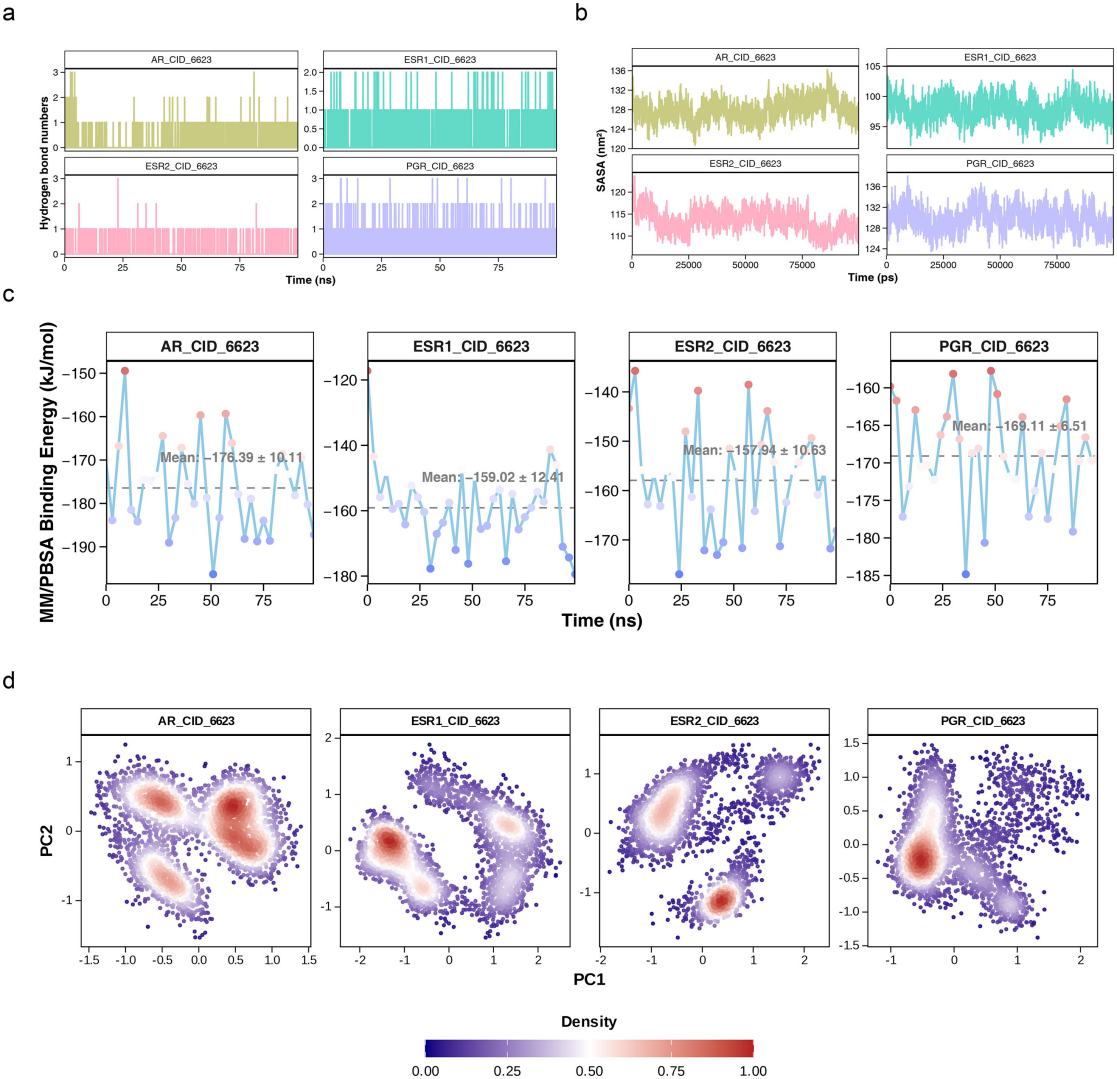
Hydrogen bond occupancy, SASA, MM/PBSA binding enthalpy, and PCA of BPA–protein complexes over 100 ns. **(a)** Number of hydrogen bonds formed between BPA and AR, ESR1, ESR2, and PGR over simulation time. **(b)** Solvent-accessible surface area (SASA) of the BPA–AR, BPA–ESR1, BPA–ESR2, and BPA–PGR complexes over simulation time. **(c)** MM/PBSA binding enthalpy (ΔH) of the BPA–AR, BPA–ESR1, BPA–ESR2, and BPA–PGR complexes. **(d)** PCA of the Cα-atom trajectories of AR, ESR1, ESR2, and PGR in complex with BPA, projected onto the first two principal components (PC1 and PC2). BPA, bisphenol A; AR, androgen receptor; ESR1, estrogen receptor alpha; ESR2, estrogen receptor beta; PGR, progesterone receptor; SASA, solvent-accessible surface area; MM/PBSA, molecular mechanics Poisson–Boltzmann/generalized Born surface area; PCA, principal component analysis; PC, principal component.

To further evaluate the stability of the four BPA–protein complexes, hydrogen bond occupancy, SASA, MM/PBSA binding enthalpy, and PCA-based conformational analyses were performed. The BPA–ESR1 complex maintained **Supplementary Tables 1**, **2** hydrogen bonds throughout the 100 ns simulation, whereas BPA–AR and BPA–ESR2 showed frequent fluctuations with transient loss of hydrogen bonds, and BPA–PGR formed 2–3 hydrogen bonds with moderate variability **Figure 9a**). SASA analysis showed overall equilibrium fluctuations without long-term trends. BPA–ESR1 exhibited the lowest SASA (95–105 nm^2^) and smallest fluctuation, indicating a compact structure, while BPA–AR showed the highest SASA (124–132 nm^2^) with large variability. BPA–ESR2 (108–122 nm^2^) and BPA–PGR (124–136 nm^2^) displayed intermediate-to-high exposure with greater fluctuations **Figure 9b**). MM/PBSA results indicated favorable binding for all complexes. BPA–AR showed the most negative mean ΔH (−179.38 ± 10.11 kcal/mol) but with noticeable fluctuations (~10 and 50 ns), followed by BPA–ESR2 (−174.84 ± 10.63 kcal/mol). BPA–PGR exhibited a moderately weaker but more stable ΔH (−163.11 ± 6.51 kcal/mol), whereas BPA–ESR1 showed the least negative value (−159.02 ± 13.43 kcal/mol) with the highest variability **Figure 9c**). PCA revealed distinct conformational landscapes: BPA–PGR formed a single dominant cluster, indicating a stable conformation; BPA–AR showed multiple dispersed clusters, reflecting high conformational heterogeneity; and BPA–ESR1/ESR2 each exhibited two main clusters, indicating intermediate flexibility with ESR2 adopting more compact conformations than ESR1 **Figure 9d**). Collectively, these analyses complement the RMSD, RMSF, and Rg results, demonstrating stable binding of BPA to all receptors while revealing receptor-dependent differences in binding energetics and conformational dynamics.

## 4 Discussion

In this study, we utilized a comprehensive approach to identify 99 potential target genes linked to BPA‐induced PP through data mining from ChEMBL, STITCH, Swiss Target Prediction, CTD, GeneCards and OMIM databases. Using the STRING database and Cytoscape software, we constructed an interaction network of these targets and identified four candidate genes: *AR*, *ESR1*, *ESR2* and *PGR*, which were recognized as important targets in BPA–induced PP toxicity. GO and KEGG were conducted to explore their potential roles in PP, which also provided new insights into PP diagnosis and treatment. Finally, molecular docking of these main targets revealed that BPA’s binding affinity to these targets may lead to adverse effects. These findings suggest that BPA is associated with sex hormone balance disruption through interactions with these genes, which could potentially contribute to PP development. However, this hypothesis requires experimental confirmation. Compared with prior BPA computational studies, our results corroborate the reported BPA–ERRγ binding (9) and extend the literature by focusing on PP. Unlike cancer/metabolic studies, we integrate *AR*, *ESR1*, *ESR2*, and *PGR* within a network toxicology–MD framework, providing a more systematic mechanistic view.

Chen et al. (24) analyzed the BPA concentrations in the urine of 136 school-age girls with idiopathic CPP (ICPP) and 136 healthy girls, and found that the BPA concentration in the urine of the ICPP group was significantly higher than that of the control group; the highest concentrations were associated with a 9.08-fold increased risk of ICPP (24). These findings suggest that BPA exposure is associated with an elevated risk of ICPP in school-aged girl (17, 24). These findings support the association between BPA and the potential target genes of PP identified in our study, further supporting the plausibility of the association between BPA exposure and PP development. Our study identified four target genes: *AR*, *ESR1*, *ESR2* and *PGR*, which are core members of the steroid hormone receptor family, and play a key role in the endocrine system. Their dysfunction directly affects sex hormone balance, especially in male and female reproductive system diseases (25, 26).

The androgen receptor (*AR*) is encoded by a gene located on the X chromosome that provides the necessary genetic instructions for synthesizing the androgen receptor protein. Androgen receptors are present in numerous tissues throughout the body, where they bind to androgens. The resulting androgen receptor complex subsequently binds to DNA and regulates the activity of specific genes involved in male sexual development while participating in sperm production, promotes the appearance of secondary sexual characteristics in males during adolescence, and also participates in regulating muscle mass and bone density in nonreproductive tissues (27). Additionally, *AR* gene abnormal function or expression changes may affect the androgen signaling pathway, thereby interfering with the normal function of the hypothalamus–pituitary–gonadal axis. This axis is the core endocrine system regulating puberty development. Disrupting this axis may lead to the abnormal initiation of puberty, causing PP or other sexual development abnormalities (28). Bermúdez de la Vega et al. reported a family with four members affected by complete androgen insensitivity syndrome, one of whom developed central PP associated with an *AR* gene mutation (3). These observations suggest that alteration in *AR*-mediated androgen signaling is involved in the regulation of GnRH secretion and could represent one of the pathways linking BPA exposure to PP, pending experimental verification.

The *ESR1* gene on chromosome 6q25 encodes ER α, which is widely distributed in female tissues such as the breast, uterus, and ovaries (29). As a member of the nuclear receptor superfamily—a group of ligand-inducible transcription factors—*ESR1* is responsible for mediating the physiological effects of estrogens (30). It promotes the development of secondary sexual characteristics in females during adolescence, participates in bone growth, maintains bone density, and protects the cardiovascular system (31). Li et al. reported a case where a novel mutation of the ER gene was detected in girls with PP (32). Of the many polymorphic sites of ER genes, the most frequently investigated polymorphisms are PvuII (397T4C, rs2234693) plus XbaI (351G4A, rs9340799) polymorphisms for ESR α. The meta-analysis study conducted by Luo revealed that *ESR1* PvuII polymorphism is associated with risk of PP for the recessive model (33). Ke et al. identified a significant association between *ESR1* XbaI polymorphism and PP (34). However, the PvuII and XbaI polymorphisms in the Rea gene may not be associated with PP development (35, 36). ESR1–PP inconsistencies may stem from context-dependent intronic variants, limited power, and differences between CPP and peripheral PP. Our results identify ESR1 as a BPA–PP hub, suggesting ligand-induced receptor dysfunction distinct from genetic variation, warranting further validation.

The *ESR2* on chromosome 14q23–24 with a total size of 40 kb encodes the ER β, which is a 530 amino acid protein (37), and its function is different from that of *ESR1*. *ESR2* affects the growth and survival of cancer cells by regulating the expression of genes related to cell proliferation and apoptosis (38, 39). The common mutation sites of the *ESR2* gene are the RsaI (1082G4A, rs1256049) and AluI (1730G4A, rs4986938) polymorphisms (40). The study showed that children with *ESR2* RsaI polymorphism were more susceptible to environmental endocrine disruptors (41), and more susceptible to PP, which corroborates the hypothesis suggested by our computational findings. However, at present, only a few studies have explored the relationship between the *ESR2* gene and PP, so further clinical and animal experimental studies are needed. BPA-related PP shows sex differences (2), with a higher incidence in girls likely due to estrogen-axis sensitivity. BPA may promote GnRH activation via ESR1/ESR2 in girls and exert context-dependent AR effects in boys. Our results suggest receptor-specific sex-divergent mechanisms that require sex-stratified validation.

The progesterone receptor (*PGR*) gene is located on chromosome 11 and encodes progesterone receptors, which are divided into two subtypes—PRA and PRB—and regulates the expression of target genes by binding to progesterone (42). PGR plays various roles in reproductive tissues and thus coordinates mammalian fertility. The transcriptional mechanism of *PGR* can promote ovulation (43).The genetic variation in the *PGR* gene may trigger preterm labor (44). Only a few studies have explored the direct correlation between *PGR* and PP, but *PGR* gene polymorphisms (such as the PROGINS insertion-deletion variation) are associated with reproductive system diseases, such as preterm birth and endometriosis (45). Although evidence linking *PGR* to PP remains limited, BPA–induced *PGR* disruption has been implicated in reproductive dysfunction. Ghosh et al. showed that BPA alters follicular *PGR* expression and localization in zebrafish, impairing ovulation, and reported that BPA–PGR binding is stable (46). Compared with experimental reproductive models, this study extends BPA–*PGR* analysis to a human PP context using network toxicology, docking, and MD, providing a complementary hypothesis linking reproductive toxicity to pubertal dysregulation.

From a physiological perspective, pubertal onset is governed by hypothalamic GnRH pulse generator activity guided by upstream regulators, including MKRN3, TAC3, and the KISS1/KISS1R system (47, 48). Notably, classical central regulators, such as MKRN3, were not identified in this study, possibly reflecting database bias toward peripheral reproductive tissues rather than hypothalamic targets. Among the identified genes, *ESR1* is well established to be expressed in hypothalamic kisspeptin neurons, while *AR*, *ESR2*, and *PGR* are present in hypothalamic reproductive regulatory circuits, collectively suggesting a potential role in modulating *KISS1*–*GnRH* signaling. BPA may therefore disrupt steroid hormone signaling within this network, leading to dysregulated *KISS1* expression and altered *GnRH* pulsatility.

First, through toxicity prediction, we found that although BPA has relatively low acute oral toxicity in specific activity tests, such as on targets like cytochrome (CYP2C9) and (CYP2E1), BPA shows a relatively high probability of predicted activity. Xu et al.’s (49) study showed the induction effect of BPA on gene expression and enzyme activity of CYP2C9. Another study showed that ATX prevents BPA–induced nephrotoxicity by inhibiting P450 CYP2C9 (50). These results corroborate ours and align with known endocrine-disrupting properties. BPA may potentially interact with estrogen receptor-related signaling pathways in a manner potentially relevant to PP pathogenesis. Experimental validation is needed to confirm these associations. BPA shows non-monotonic dose-response effects, with low-dose receptor-mediated activity. Despite low acute toxicity (LD50 4,950 mg/kg), epidemiological studies link low-level exposure to ICPP. Docking (−7.6 to −8.5 kcal/mol) suggests receptor binding but requires dose-response validation.

The molecular docking results indicate a strong binding affinity between BPA and the proteins encoded by the key genes, and it showed a stable binding state during the simulation process. These results provide computational evidence for potential interactions between BPA and proteins such as estrogen receptors; they also provide molecular-level evidence for BPA’s interference with the endocrine system (49, 51). In addition, molecular dynamics simulations revealed the dynamic process of the interaction between BPA and these proteins, providing preliminary computational insights into how BPA may potentially influence cell function and signal transduction.

Although this study provides molecular-level insights into BPA effects on PP development, some limitations exist. First, the research is mainly based on bioinformatics analysis and computational simulation, and has not yet been verified through experimental studies. Further *in vitro*, *in vivo*, receptor-based, and multi-omics studies are required to verify the identified targets and mechanisms. Second, the target databases and PP-related gene sets may not fully capture all relevant biological information. Finally, the computational and cross-sectional nature of this study limits causal inference; therefore, the findings should be considered hypothesis generating rather than mechanistically conclusive. In addition, each BPA–protein complex had one 100 ns MD trajectory without replicates. No cross-run convergence statistics were conducted; stability and energy data represent only a single simulation. Replicate MD runs are required for robust convergence and binding energy validation in future work. Future studies should include multi-level experimental validation to substantiate the computational findings, including *in vitro* assays in ovarian granulosa and hypothalamic neuronal cells to assess BPA–induced regulation of *AR*, *ESR1*, *ESR2*, and *PGR*; *in vivo* rodent exposure models to evaluate reproductive outcomes and hypothalamic–pituitary–gonadal axis activity; receptor-based luciferase reporter assays to confirm nuclear receptor activation; and transcriptomic and proteomic analyses (e.g., qPCR and Western blotting) to validate gene and protein-level changes after BPA exposure.

## 5 Conclusions

Through network toxicology and molecular dynamics simulation methods, this study deeply explored the potential impact of BPA on PP and its molecular mechanism. A total of 99 target genes related to BPA’s effects on PP were identified, and the PPI network further identified *AR*, *PGR*, *ESR1* and *ESR2* as key genes. The computational results suggest that BPA interacts with multiple key genes involved in endocrine regulation, and that its binding to estrogen receptors could potentially be associated with disrupted sexual development. These findings provide candidate molecular targets for future experimental investigations into the relationship between BPA exposure and PP. In addition, the research revealed the binding affinity between BPA and PP-related gene products, providing candidate molecular targets for future experimental investigation.

## Data Availability

The original contributions presented in the study are included in the article/**Supplementary Material**. Further inquiries can be directed to the corresponding authors.

## Glossary

PP: Precocious puberty
BPA: Bisphenol A
GO: Gene ontology
KEGG: Kyoto Encyclopedia of Genes and Genomes
PPI: Protein–protein interaction
CPP: Central pp
HPG: Hypothalamic–pituitary–gonadal
PPP: Peripheral pp
EDC: Endocrine disruptor
SMILES: Simplified molecular input line entry system
STITCH: Search tool for interacting chemicals
CTD: Chemical toxicity database
UniProt: Universal protein resource
OMIM: Online Mendelian Inheritance in Man
LD50: Lethal dose
BP: Biological process
CC: Cellular component
MF: Molecular function
PDB: Protein data bank
RMSD: Root mean square deviation
RMSF: Root mean square fluctuation
ER: Estrogen receptor
ICPP: Idiopathic cpp
AR: Androgen receptor
PGR: Progesterone receptor
